# An Optical Fiber Refractive Index Sensor Based on the Hybrid Mode of Tamm and Surface Plasmon Polaritons

**DOI:** 10.3390/s18072129

**Published:** 2018-07-03

**Authors:** Xian Zhang, Xiao-Song Zhu, Yi-Wei Shi

**Affiliations:** 1 School of Information Science and Engineering, Fudan University, 220 Handan Rd, Shanghai 200433, China; xianzhang16@fudan.edu.cn (X.Z.); ywshi@fudan.edu.cn (Y.-W.S.); 2 Key Laboratory for Information Science of Electromagnetic Waves (MoE), Fudan University, 220 Handan Rd, Shanghai 200433, China

**Keywords:** Tamm plasmon polariton, surface plasmon polariton, one dimensional photonic crystal, refractive index, fiber sensor

## Abstract

A novel high performance optical fiber refractive index (RI) sensor based on the hybrid transverse magnetic (TM) mode of Tamm plasmon polariton (TPP) and surface plasmon polariton (SPP) is proposed. The structure of the sensor is a multi-mode optical fiber with a one dimensional photonic crystal (1 DPC)/metal multi-films outer coated on its fiber core. A simulation study of the proposed sensor is carried out with the geometrical optical model to investigate the performance of the designed sensor with respect to the center wavelength, bilayer period and the thickness of silver layer. Because the lights transmitted in the fiber sensor have much larger incident angles than those in the prism based sensors, the center wavelength of the 1 DPC should shift to longer wavelength. When the coupling between TM-TPP and SPP is stronger, the sensor exhibits better performance because the electromagnetic field of the TPP-SPP hybrid mode is enhanced more in the analyte. Compared to most conventional fiber surface plasmon resonance sensors, the figure of merit of the proposed sensor is much higher while the sensitivity is comparable. The idea of utilizing TPP-SPP hybrid mode for RI sensing in the solid-core optical fiber structure presented in this paper could contribute to the study of the fiber RI sensor based on TPP.

## 1. Introduction

Surface electromagnetic waves, including Bloch surface wave (BSW) excited at the interface between a homogeneous medium and a finite one-dimensional photonic crystal (1 DPC) and surface plasmon polariton (SPP) excited at the interface between metal and dielectric, have been applied to biochemical sensing in recent years [1,2,3,4]. Excitation of BSW and SPP requires phase-matching for the tangential component of the wave factor, which can be satisfied under total internal reflection condition. Tamm plasmon polariton (TPP) is another surface mode which can be excited through the interaction of light at the boundary of the Bragg mirror heterostructures or the one between metal and dielectric Bragg mirror [5,6]. TPPs exist for both transverse magnetic (TM) and transverse electric (TE) polarizations [7]. It has been studied in many applications such as the development of diodes and lasers [8], the generation of efficient third harmonic [9], the enhancement of nonlinearity for devising low threshold bistable optical switches [10,11], the realization of efficient magneto-photonic devices [12,13] and the development of monolithically-integrated photonic components and sensors [14,15,16].

Contrary to BSW and SPP, excitation of TPP does not require phase-matching for the tangential component of the wave factor and can be realized at any incident angle [5]. Studies have shown that TPP demonstrates itself as a narrow absorption resonance dip in the reflectance spectrum of 1 DPC/metal system [17]. However, the electromagnetic field of TPP on the 1 DPC/metal interface rarely extended into the surrounding analyte on the other side of the metal film like BSW and SPP. It is difficult to directly utilize TPP for sensing applications like surface plasmon resonance (SPR) sensors. In a 1 DPC terminated by a thin plasmonic metal film, TPP and SPP can be excited simultaneously when total internal reflection condition is satisfied. Coupling between TM-TPP at the 1 DPC/metal interface and SPP at the metal/analyte interface might lead to the appearance of the TPP-SPP hybrid mode [18,19]. The electromagnetic field of TPP localizes at the boundary of 1 DPC and metal, while that of SPP gets a huge enhancement at the metal/analyte interface and decays exponentially away from it. As a result, the electromagnetic field of the hybrid mode is enhanced on the metal/analyte interface and extends into the analyte medium. Therefore, the TPP-SPP hybrid mode has large potential for refractive index (RI) sensing [20,21]. However, to our knowledge, there is no report on the sensing application of TPP-SPP hybrid mode based on solid-core fiber structure.

In this paper, a high performance solid-core fiber refractive index sensor utilizing TPP-SPP hybrid mode excited in the 1 DPC/metal structure is proposed. Theoretical analysis with a geometrical optical model is carried out to investigate the characteristics of the designed sensor. The interaction between TM-TPP and SPP is studied. We have optimized different parameters such as thickness of the metal layer, bilayer period and center wavelength of the band gap. The results show that the RI detection range of the designed sensor with optimal parameters is extended compared with the conventional solid-core fiber SPR sensors, while the figure of merit (FOM) is much higher.

## 2. Sensor Configuration and Theoretical Model

**Figure 1:**
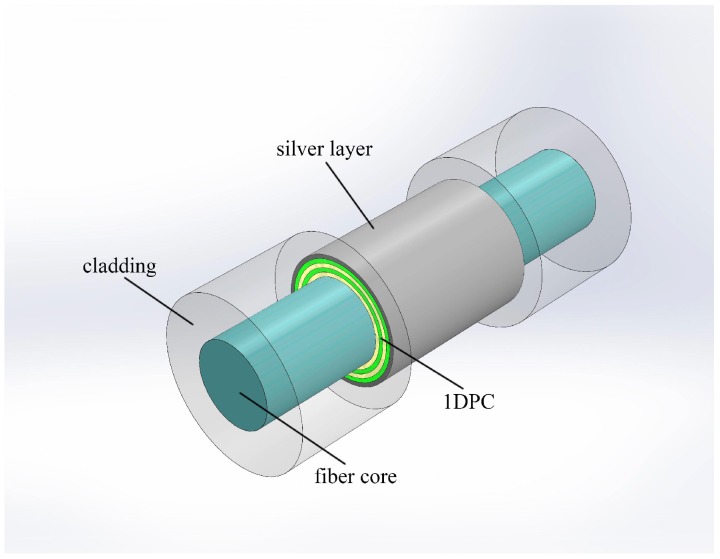
Structure of the solid-core fiber RI sensor based on 1 DPC/silver system.

Figure 1 shows the proposed solid-core fiber refractive index sensor based on 1 DPC/metal structure. After removing the cladding of a multi-mode optical fiber (MMF) for a short length that can be done by chemical etching with hydrofluoric acid, periodically bilayers of TiO_2_/SiO_2_ are coated on the surface of the fused silica fiber core. Then the periodically bilayers are outer covered by a thin silver film. Atomic layer deposition (ALD) technology is the ideal method for the deposition of the 1 DPC/metal multilayers. It has been proven to fabricate high quality 1 DPC on the outer surface of solid-core fiber [22]. To satisfy the condition of total reflection, the RI of analyte outside the fiber should be lower than that of the fiber core. TM-TPP and SPP would be excited simultaneously and interact with each other when appropriate light transmits in the MMF. Consequently, TPP-SPP hybrid mode will demonstrate itself as a narrow resonance dip located in the band gap of 1 DPC in the transmission spectrum.

The diameter of the fiber core is 200 μm and numerical aperture (NA) is 0.2. The 1 DPC structure is comprised of periodic multilayers of TiO_2_/SiO_2_, with RI *n*_1_ = 2.45 and *n*_2_ = 1.46 approximately, respectively. These two kinds of oxides have been widely used for 1 DPC in many applications [10,20]. The thickness of the two layers is denoted by *d*_1_ and *d*_2_, which is given by the following quarter-wave film system [23]:

(1) $$n_{1}d_{1}=n_{2}d_{2}=\lambda_{0}/4.$$

where *λ*_0_ is the center wavelength of the 1 DPC band gap. Silver is chosen as the metal material since it is well known as a good candidate for the SPR sensor. The dielectric constant of the silver layer is obtained by the following Drude free electron model [24]:

(2) $$\varepsilon (\lambda )=\varepsilon_{r}+i\varepsilon_{i}=1-\frac{\lambda^{2}\lambda_{c}}{\lambda_{p}^{2}(\lambda_{c}+i\lambda )}.$$

where *λ_p_* = 1.4541 × 10^−7^ m, *λ_c_* = 1.7614 × 10^−5^ m.

**Figure 2:**
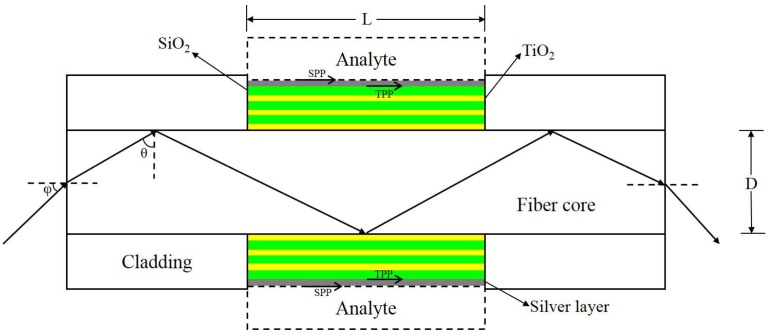
Sketch and ray model of solid-core fiber refractive index sensor based on 1 DPC/silver structure.

Since the diameter of fiber core is much larger than the wavelength of input light, a geometrical optical model illustrated in Figure 2 is employed to theoretically calculate the transmission spectrum of the proposed sensor [25]. Here we only consider the meridional rays. Here we assume the distribution of the input light power is close to Gaussian with profile *P_in_*(φ) expressed approximately as [26]:

(3) $$P_{in}(\varphi )\propto \exp(-\varphi^{2}/\varphi_{0}{\left(\lambda \right)}^{2}).$$

where φ is the launching angle of the incident light, φ_0_ depends on the input light source. The relationship between angle *θ* and φ shown in Figure 2 follows Snell’s law sin(φ) = *n*cos(*θ*), where *n* is the RI of the fiber core material.

The power of the output light of the sensor at a given wavelength is given as:

(4) $$P_{out}=\int_{\theta_{cr}}^{\pi /2}P_{in}(\theta )R_{p}{\left(\theta \right)}^{N(\theta )}d\theta .$$

Where *θ_cr_* denotes the critical angle of total reflection on the interface between fiber core and cladding, which is approximately 82.1° for NA = 0.2. *θ* denotes the angle between the normal of the reflection interface and the direction of the incident light. *R_p_*(*θ*) is the reflectance for the p-polarized light on the surface of fiber core which is calculated by the transfer matrix method [27]. Reflection times of the light *N*(*θ*) is a function of *θ*, the length of the sensitive area *L* and core diameter *D*, which is:

(5) $$N(\theta )=\frac{L}{D\tan\theta }.$$

Therefore, the generalized expression for the transmittance of the sensor can be expressed as:

(6) $$T=\frac{\int_{\theta_{cr}}^{\pi /2}P_{in}(\theta )R_{p}{\left(\theta \right)}^{N(\theta )}d\theta }{\int_{\theta_{cr}}^{\pi /2}P_{in}(\theta )d\theta }.$$

With Equation (6), the performance of the proposed sensor could be numerically evaluated. While TM-TPP and SPP are excited simultaneously and interact with each other, the electric field located on the 1 DPC/Ag interface is extended into the analyte, leading to a TPP-SPP hybrid mode. It would exhibit a minimum in the transmission spectrum at a particular wavelength located in the band gap of 1 DPC, known as the resonance wavelength (RW). The RW will shift when the RI of the analyte changes. Therefore, by measuring the RW from the transmission spectra of the proposed sensor, the RI of the analyte can be obtained.

Sensitivity is an important parameter to evaluate the performance of a SPR sensor. Usually, the sensitivity of a wavelength-interrogated SPR sensor is defined as:

(7) $$S=\frac{\Delta \lambda_{res}}{\Delta n_{s}},$$

where Δ*λ_res_* is the shift in the RW due to the RI change of analyte Δ*n_s_*. How accurately the value of RW can be determined depends on the full width at half maximum (FWHM) of the resonance dip in the transmission spectrum. The overall performance of a sensor is defined in terms of FOM as:

(8) $$FOM=\frac{S}{FWHM}.$$

## 3. Results and Discussion

**Figure 3:**
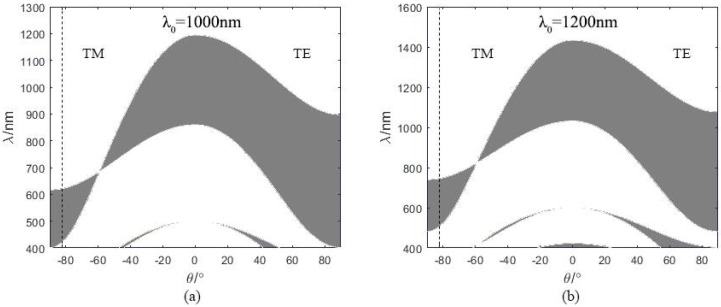
Band gap of 1 DPC with different center wavelength. The left side represents p-polarized light, while the right side represents s-polarized light. (**a**) *λ*_0_ = 1000 nm, (**b**) *λ*_0_ = 1200 nm.

Center wavelength *λ*_0_ is the primary parameter for designing 1 DPC. It implies where the band gap for the normal incident light is located. However, most of the input lights transmit with large incident angles in the optical fiber as shown in Figure 2. Therefore, the band gap of TM light with large incident angles near 90° instead of small incident angles is more meaningful to the designed fiber sensor. Figure 3 shows the band gap of 1 DPC with different center wavelength, which is calculated by the methods reported in [27]. The left half and right half represents p-polarized and s-polarized light, i.e., TM and TE light, respectively. The shading represents wavelengths that are forbidden to propagate in the 1 DPC. The band gap locates around the center wavelength only in small incident angle ranges and it shifts towards shorter wavelength when the incident angle increases. We firstly adopt *λ*_0_ = 1000 nm to investigate the transmission spectrum of the proposed sensor. As can be seen in Figure 3a, the band gap of the 1 DPC at incident angle larger than 82.1° nearly covers the wavelength range of 400–600 nm, which is around the SPR RW when analyte is water. Figure 3b shows the band gap of the 1 DPC with 1200 nm center wavelength. It can be observed that the band gap shifts towards longer wavelength with the increase of the center wavelength.

**Figure 4:**
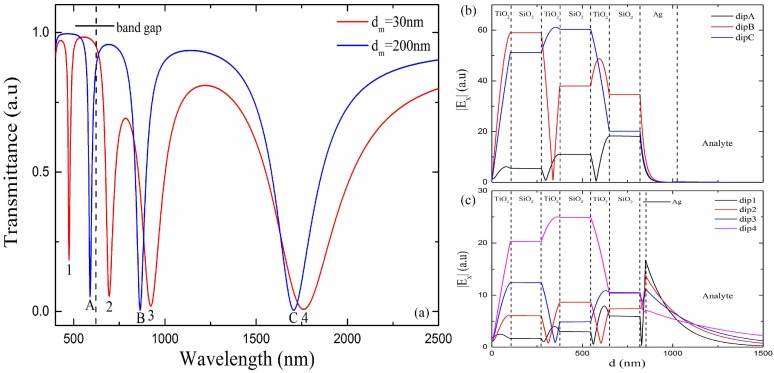
(**a**) Transmission spectra of the proposed sensor with different thickness of silver layer at *n_s_* = 1.37, *N* = 3, *λ*_0_ = 1000 nm. (**b**) Tangential electric field distribution at the resonance wavelength 590 nm and the resonance angle of 87.25°, 864 nm and 89.54°, 1706 nm and 89.64° for dips A, B and C, respectively. The thickness of silver layer is 200 nm. (**c**) Tangential electric field distribution at 474 nm and 87.77°, 694 nm and 87.01°, 923 nm and 87.88°, 1761 nm and 89.11° for dips 1 to 4, respectively. The thickness of silver layer is 30 nm.

The length of the sensitive area *L* affects the reflection times of the transmission light on the sensing surface. The depth of resonance dip increases with larger *L*. However, when the length is too long, the increase of the depth of resonance dip becomes negligible and the sensor becomes fragile. Therefore, we adopt *L* = 5 cm in all following calculations. The thickness of silver layer *d_m_* should be small enough to excite SPP. Figure 4a shows the transmission spectra of the designed sensors with 30 nm and 200 nm silver layer thicknesses when p-polarized light is incident. The bilayer period N adopted in the calculation is three. We also calculated the electric field distribution to investigate the origin of the multiple resonance dips shown in Figure 4a. The resonance dips are marked as dip 1 to 4 and dip A to C from short to long wavelength for *d_m_* = 30 nm and 200 nm, respectively. Figure 4b and 4c show the distributions of the tangential electric field amplitude at these resonance wavelengths. As shown in Figure 4b, none of the three resonance dips is caused by SPR because the 200 nm silver layer is too thick. Dip A, which is located in the band gap of 1 DPC, is caused by TM-TPP. Its electric field reaches maximum near the 1 DPC/Ag interface and decays in an oscillating form away from it in the 1 DPC. The electric field distributions of dips B and C outside the band gap indicate that these two dips are ascribed to the waveguide modes in the 1 DPC. There are almost no electric field distribution in the analyte for all three modes. When the silver layer is thin, the TM-TPP and SPP are excited simultaneously and interact with each other. The different distributions of electric field of the four resonance dips shown in Figure 4c indicate their different origins. Dip 1 located in the band gap is caused by the TPP-SPP hybrid mode. Its electric field is a typical TPP mode in the 1 DPC, while it is enhanced obviously at the Ag/analyte interface and decays exponentially away from it. Such kind of electric field indicates the coupling of the TM-TPP and SPP. The electric field in 1 DPC and enhancement at the silver/analyte interface indicate that dips 2 and 3 are ascribed to the coupling between SPP and waveguide modes in the 1 DPC. Dip 4 is almost caused by pure waveguide mode since there is nearly no enhancement at the Ag/analyte interface. Thus, the silver layer should be thin enough to excite the TPP-SPP hybrid mode, which can extend its electric field distribution into the analyte for RI sensing.

**Figure 5:**
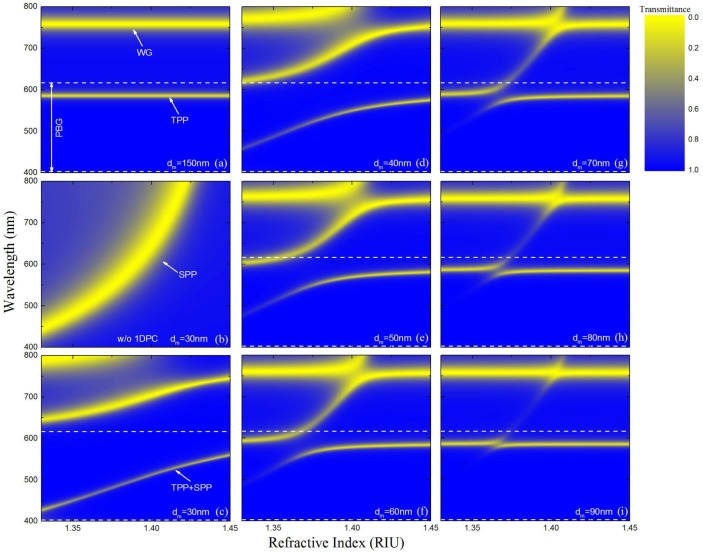
Two-dimensional contour maps of normalized transmittance calculated through the proposed sensor as a function of wavelength and RI of the sensed medium at *N* = 3, *λ*_0_ = 1000 nm. (**a**) *d_m_* = 150 nm, (**b**) without 1 DPC, *d_m_* = 30 nm, (**c**) *d_m_* = 30 nm, (**d**) *d_m_* = 40 nm, (**e**) *d_m_* = 50 nm, (**f**) *d_m_* = 60 nm, (**g**) *d_m_* = 70 nm, (**h**) *d_m_* = 80 nm, (**i**) *d_m_* = 90 nm.

The thickness of silver layer affects the strength of the interaction between TPP and SPP excited at the 1 DPC/Ag interface and Ag/analyte interface, respectively. Figure 5 shows the two-dimensional contour maps of the normalized transmittance calculated through the proposed sensor as a function of wavelength and RI of the sensed medium when p-polarized light is incident. Here the bilayer period *N* = 3, center wavelength *λ*_0_ = 1000 nm. The white dashed lines indicate the photonic bandgap (PBG). The yellow regions represent the resonance dips in the transmission spectra. Only TPP is excited in the 1 DPC/metal structure when the silver layer is too thick to penetrate as shown in Figure 5a. Here the silver layer thickness is 150 nm. The dip located near 750 nm is the waveguide mode (WG). The resonance dip of TPP does not shift when the RI of the surrounding analyte changes. Figure 5b shows the normalized transmittance of the sensor without the 1 DPC structure. In this case, the sensor is just a conventional solid-core fiber SPR sensor with a single 30 nm silver layer. The SPR dip in the figure red-shifts as the sensed RI increases. It can be observed that the width of TPP dip is much smaller than that of SPR dip, which is beneficial to the measurement precision. When the silver layer is thin enough, TPP and SPP are simultaneously excited and interact with each other. They demonstrate themselves as two repulsive resonance dips. The repulsion becomes weaker with the increasing silver layer thickness, which is indicated by the decreasing distance between the two resonance dips as shown in Figure 5c–i.

**Figure 6:**
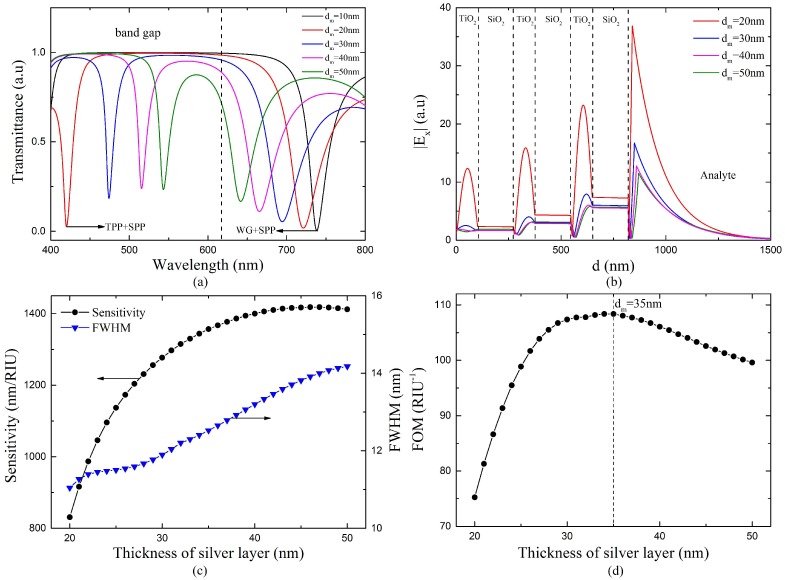
(**a**) Transmission spectra of the proposed sensor with different thickness of silver layer at *n_s_* = 1.37, *N* = 3, *λ*_0_ = 1000 nm. (**b**) Tangential electric field distribution of TPP-SPP dip at the resonance wavelength 420 nm and the resonance angle of 88.63°, 474 nm and 87.77°, 516 nm and 87.43°, 543 nm and 87.24° for *d_m_* = 20 nm, 30 nm, 40 nm, 50 nm, respectively. (**c**) The variations of sensitivity and FWHM of the proposed sensor versus the silver layer thickness at *n_s_* = 1.37. (**d**) The variation of FOM versus the silver layer thickness at *n_s_* = 1.37.

The slope of the variation of the TPP-SPP hybrid mode resonance wavelength in Figure 5 represents the sensitivity of the sensor. It can be seen that the sensitivity of the sensor degrades rapidly when the silver layer thickness is above 50 nm. Thus, we will further analyze the performance of the sensor with different silver layer thickness below 50 nm to obtain the optimal thickness. Figure 6a shows the transmission spectra of the proposed sensors with different silver layer thicknesses. Here the RI of the analyte *n_s_* = 1.37, the bilayer period *N* = 3, center wavelength *λ*_0_ = 1000 nm. As shown in the figure, the two resonance dips get closer and overlap when the thickness of the silver layer increases. This is because the repulsing coupling between the TM-TPP and SPP weakens as the silver thickness increases [18]. The electric fields of the TPP-SPP hybrid modes with different *d_m_* shown in Figure 6b also prove this. As the silver layer thickness increases, the enhancement at the Ag/analyte interface becomes weaker, which indicates the coupling between TM-TPP and TPP weakens. However, the silver layer with a thickness less than 30 nm will cause the resonance dip excited by TPP-SPP hybrid state to shift towards ultraviolet range, where the fused silica fiber is not transparent. Figure 6c and d show the performance of the proposed sensor with various thickness of the silver layer at *n_s_* = 1.37. Results show that the FWHM increases with the thickness of silver layer, while the FOM reaches its maximum value at *d_m_* = 35 nm. Therefore, we adopt 35 nm as the optimal thickness of the silver layer.

**Figure 7:**
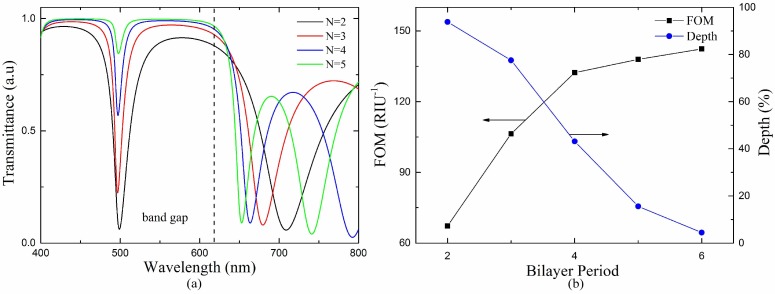
(**a**) Transmission spectra of the proposed sensor with different bilayer periods at *n_s_* = 1.37, *λ*_0_ = 1000 nm, *d_m_* = 35 nm. (**b**) The depth of resonance dip and FOM of the proposed sensor with respect to various bilayer period at *n_s_* = 1.37.

The transmission spectra of the proposed sensor with different bilayer periods are shown in Figure 7a. It can be observed that both width and depth of the TPP-SPP resonance dip decrease when the bilayer period increases. As the bilayer period increases, the more perfect 1 DPC and the stronger restriction of the band gap makes the resonance dip narrower. On the other hand, the thicker 1 DPC structure makes the excitation of the TPP-SPP hybrid mode more difficult, which shallows the resonance dip. As defined by Equation (8), the FOM is inversely proportional to the FWHM. Therefore, the FOM increases with the bilayer periods. However, the depth of resonance dip should not be too small for better detecting. Hence, there is a trade-off between the width and depth of the resonance dip. We calculate the performance of the proposed sensor and find that the sensitivity changes little with the bilayer period, which is always in the range of 1310–1420 nm/RIU. However, the FOM and dip depth change a lot. Figure 7b shows the depth of resonance dip and the FOM of the proposed sensor with different bilayer period at *n_s_* = 1.37. It can be seen that the FOM increases rapidly before *N* = 4 and much slower after that, while the depth of resonance dip decreases consistently. When the bilayer period increases from 4 to 5, the FOM increases slightly while the depth of resonance dip decreases dramatically from 0.43 to 0.15. Therefore, we adopt four pairs of bilayer as the optimal bilayer period.

**Figure 8:**
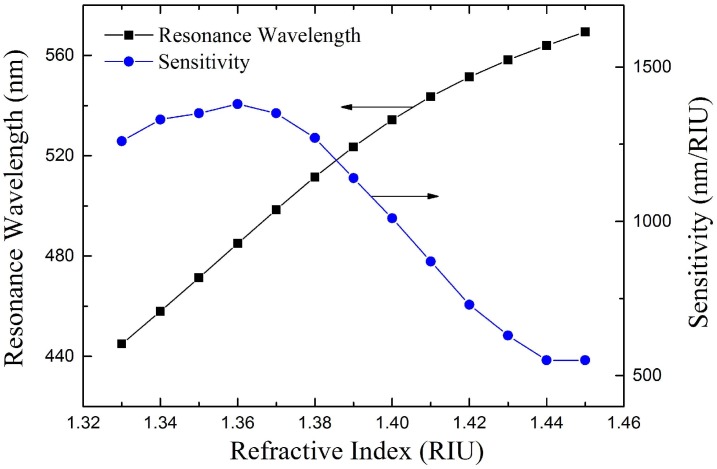
Theoretical results of resonance wavelength and sensitivity of the proposed sensor at *λ*_0_ = 1000 nm, *N* = 4, *d_m_* = 35 nm.

Then we will analyze the performance of the proposed sensor with the optimal structure parameters obtained above, which are bilayer period *N* = 4, center wavelength *λ*_0_ = 1000 nm, the thickness of silver layer *d_m_* = 35 nm. The RWs are obtained from the calculated transmission spectra, and the sensitivity of the sensor is calculated from the RWs by Equation (7). The variations of RW and sensitivity with respect to RI of the analyte are shown in Figure 8. The RI detection range of the proposed sensor is 1.33–1.45, which is much wider than conventional fiber SPR sensors since it can detect RI very close to the fiber core [28]. The sensitivity varies from 550 to 1380 nm/RIU in the range. However, the sensitivity decreases rapidly when the RI of analyte is larger than 1.38, which make the sensor’s performance degrades in the RI range of 1.38–1.45.

**Figure 9:**
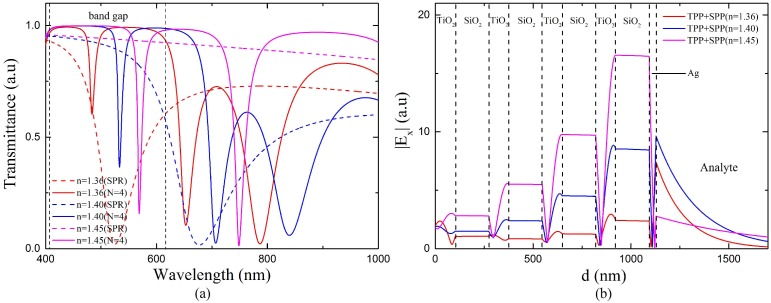
(**a**) Transmission spectra of the proposed sensor with different RI of the analyte at *N* = 4, *λ*_0_ = 1000 nm, *d_m_* = 35 nm. The transmission spectra of the sensor with a single silver layer of 35 nm is also presented for comparison. (**b**) Tangential electric field distribution of TPP-SPP dip at the resonance wavelength 484 nm and the resonance angle of 87.02°, 533 nm and 87.56°, 569 nm and 87.9° for *n_s_* = 1.36, 1.40, 1.45, respectively.

In order to investigate the cause of the decrease in sensitivity, we calculate the transmission spectra of the proposed sensor with various RI of analyte. As shown in Figure 9a, the red, blue and purple solid lines are the transmission spectra of the proposed 1 DPC/Ag sensor with sensed RI 1.36, 1.40 and 1.45, respectively. The dashed lines are the transmission spectra of a fiber SPR sensor which has the same geometry and a 35 nm silver layer outer coating but no 1 DPC structure. It can be seen that both the SPR dips of the fiber SPR sensor and the TPP-SPP dips of the proposed sensor shift towards longer wavelength as the sensed RI increases. However, the wavelength shift of the SPR dip is much larger than the TPP-SPP dip. As a result, the distance between the two dips becomes farther as the sensed RI increases, which leads to weaker interaction between TM-TPP and SPP. Figure 9b shows the amplitudes of tangential electric fields of the TPP-SPP dips at three different sensed RIs. The gradually attenuated oscillating electric filed in 1 DPC and the enhancement at the Ag/analyte interface indicate that the dips located in the band gap of 1 DPC are all caused by the excitation of the TPP-SPP hybrid mode. As the sensed RI increases, the enhancement at the Ag/analyte interface becomes weaker compared to electric field in the 1 DPC, which indicates less SPP component in the hybrid mode and weaker interaction between TM-TPP and SPP. This will make the sensor less sensitive to the variation of the analyte RI. Therefore, the sensitivity of the proposed sensor keeps decreasing when RI is above 1.37 as shown in Figure 8.

**Figure 10:**
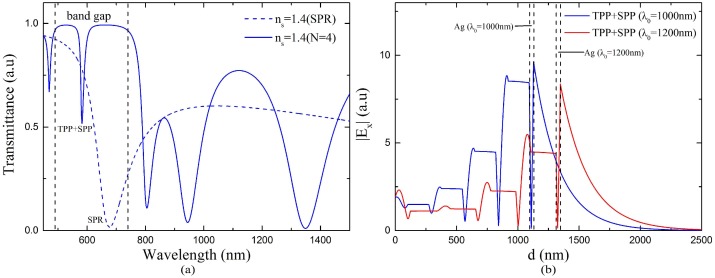
(**a**) Transmission spectrum of the proposed sensor at *N* = 4, *λ*_0_ = 1200 nm, *d_m_* = 35 nm, *n_s_* = 1.40. The transmission spectrum of the sensor with a single silver layer of 35 nm is also presented for comparison. (**b**) Tangential electric field distribution of TPP-SPP dip at the resonance wavelength 533 nm and the resonance angle of 87.56°, 582 nm and 87.45° for *λ*_0_ = 1000 nm and 1200 nm, respectively.

From the discussions above, the performance of the sensor with 1000 nm center wavelength degrades when RI is above 1.38. It might not be optimal for the whole RI range of 1.33–1.45. The proposed sensor may achieve better performance in the higher RI range by adjusting the center wavelength. As shown in Figure 9a, the SPR dip red shifts quickly while the TPP-SPP dip is always located in the band gap of 1 DPC as the sensed RI increases. The farther distance leads to weaker interaction between TM-TPP and SPP and lower sensitivities. Therefore, to strengthen the interaction, the center wavelength which decides the location of band gap of 1 DPC should be adjusted according to the RI range. We adopt the center wavelength as 1200 nm for the RI range 1.37–1.45 and the corresponding band gap of the 1 DPC is shown in Figure 3b. The transmission spectra of the fiber SPR sensor with a single 35 nm sliver layer and the proposed 1 DPC/Ag sensor are shown in Figure 10a. Here the bilayer period *N* = 4, center wavelength *λ*_0_ = 1200 nm, the thickness of silver layer *d_m_* = 35 nm, the RI of analyte *n_s_* = 1.4. As calculated by Equation (1), the thicknesses of the TiO_2_ and SiO_2_ layers are 102 and 171 nm, 122 and 205 nm for the center wavelength of 1000 nm and 1200 nm, respectively. Compared with the blue solid line in Figure 9a, the TPP-SPP resonance dip of the sensor with *λ*_0_ = 1200 nm red shifts and gets closer to the SPR dip. The tangential electric filed distributions of the TPP-SPP dips for both center wavelengths are shown in Figure 10b. The stronger enhancement at the Ag/analyte interface compared to the electric field in the 1 DPC indicates more SPP component in the hybrid mode and stronger interaction between TM-TPP and SPP with the 1200 nm center wavelength. From this point of view, 1200 nm is more suitable than 1000 nm to be set as the center wavelength of 1 DPC in the designed fiber sensor to achieve better performance in the relatively high RI range above 1.37.

**Figure 11:**
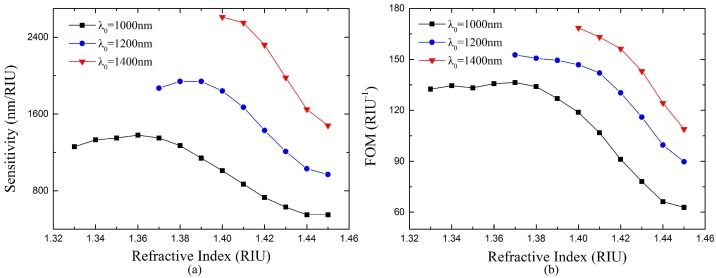
Theoretical results of performance of the proposed sensor with different center wavelength. (**a**) Sensitivity versus *n_s_*; (**b**) FOM versus *n_s_*.

Figure 11 shows the sensitivity and FOM of the proposed sensor with different center wavelength. Although the RI detection range of the sensor with *λ*_0_ = 1000 nm is 1.33–1.45, the sensitivity and FOM decrease at larger RI due to the weaker interaction between TM-TPP and SPP. Thus, the center wavelength of 1000 nm might not be suitable for the whole RI range of 1.33–1.45. Larger center wavelength leads to band gap located at longer wavelength range, which strengths the interaction between TM-TPP and SPP in the higher RI range. Therefore, the sensor with center wavelength 1200 nm has much larger sensitivities and FOMs in the RI range above 1.37. However, the lower limit of its RI detection range rises to 1.37 because the resonance dip moves out of the band gap and disappears when the RI is below 1.37. The sensor with 1400 nm center wavelength has even larger sensitivities and FOMs in the even smaller RI detection range of 1.40–1.45. From this point of view, we could adopt the appropriate center wavelength for the exact required RI range to achieve best sensor performance. A performance comparison between the proposed sensors with different center wavelengths is also shown in Table 1. The sensor with 1000 nm center wavelength has a RI detection range of 1.33–1.45. In the range below 1.38, its sensitivity and FOM are always larger than 1260 nm/RIU and 132 RIU^−1^, respectively. However, in the range above 1.38, its sensitivity and FOM gradually decrease to 550 nm/RIU and 63 RIU^−1^, respectively. In its RI detection range of 1.37–1.45, the sensor with 1200 nm center wavelength has larger sensitivity and FOM than the previous sensor. It has best performances in the RI range of 1.37–1.40 with sensitivity larger than 1840 nm/RIU and FOM larger than 146 RIU^−1^.

The sensor with 1400 nm center wavelength has even better performances than the previous two sensors in its RI range of 1.40–1.45. The FOMs of these sensors are much higher than that of most of the conventional fiber SPR sensors, which are usually below 50 RIU^−1^, while the sensitivities are comparable [29]. It is worth noting that the proposed sensor utilizing TPP-SPP hybrid mode has a large extension in the higher limit of the RI detection range which make it able to detect RI very close to the fiber core material

## 4. Conclusions

A novel solid-core fiber RI sensor with 1 DPC/Ag structure based on the TPP-SPP hybrid mode was proposed. Theoretical modelling was carried out to analyze the performance of the designed sensor. The influence of several parameters such as the center wavelength of 1 DPC, the bilayer period and the thickness of silver layer was investigated and the optimal parameter setting was obtained. The optimal center wavelength of 1 DPC changes with the RI detection range. The results show that the FOM of the proposed sensor is much higher than most of the conventional fiber SPR sensors while the sensitivity is comparable. The idea of utilizing TPP-SPP hybrid mode for RI sensing in the solid-core optical fiber structure presented in this paper could contribute to the study and design of the fiber RI sensor based on TPP.

## Figures and Tables

**Table 1:** Comparison between the proposed sensors with different center wavelengths.

Configuration	Detection RI Range RIU	Sensitivity nm/RIU	FOM RIU^−1^
*λ*_0_ = 1000 nm, *N* = 4, *d_m_* = 35 nm	1.33–1.45	550–1380	62–136
*λ*_0_ = 1200 nm, *N* = 4, *d_m_* = 35 nm	1.37–1.45	970–1940	89–152
*λ*_0_ = 1400 nm, *N* = 4, *d_m_* = 35 nm	1.40–1.45	1480–2610	108–168
